# TDP43 Exacerbates Atherosclerosis Progression by Promoting Inflammation and Lipid Uptake of Macrophages

**DOI:** 10.3389/fcell.2021.687169

**Published:** 2021-07-05

**Authors:** Ning Huangfu, Yong Wang, Zhenyu Xu, Wenyuan Zheng, Chunlan Tao, Zhenwei Li, Yewen Hu, Xiaomin Chen

**Affiliations:** Department of Cardiology, Ningbo First Hospital, Ningbo, China

**Keywords:** atherosclerosis, macrophage, inflammation, CD36, TDP43

## Abstract

**Objective:**

Atherosclerosis (AS), characterized by cholesterol overloaded-macrophages accumulation and plaque formation in blood vessels, is the major cause of cardiovascular disease. Transactive response DNA-binding protein∼43 kDa (TDP43) has recently been identified as an independent driver of neurodegenerative diseases through triggering inflammatory response. This study investigated whether TDP43 is involved in AS development, especially in macrophages-mediated-foam cell formation and inflammatory responses.

**Methods:**

Transactive response DNA-binding protein∼43 kDa expressions in oxidized low-density lipoprotein (oxLDL)-treated macrophages and peripheral blood mononuclear cells (PBMCs) from patients with coronary artery disease (CAD) were detected by real time-polymerase chain reaction (RT-PCR), Western blot, and immunofluorescence. Gene gain or loss of function was used to investigate the effects of TDP43 on macrophages-mediated lipid untake and inflammation with ELISA, protein immunoprecipitation, RT-PCR, Western blot, and immunofluorescence. Macrophage TDP43 specific knockout mice with ApoE^–/–^ background were fed with western diet for 12 weeks to establish AS model, and used to explore the role of TDP43 on AS progression.

**Results:**

Transactive response DNA-binding protein∼43 kDa expression increases in oxLDL-treated macrophages and PBMCs from patients with CAD. Furthermore, we find that TDP43 promotes activation of NF-κB to increase inflammatory factor expression in macrophages through triggering mitochondrial DNA release to activate cGAS-STING signaling. Moreover, TDP43 strengthens lipid uptake of macrophages through regulating β-catenin and PPAR-γ complex to promote scavenger receptor gene CD36 transcription. Finally, using macrophage TDP43 specific knockout mice with ApoE^–/–^ background fed with western diet for 12 weeks to establish AS model, we find that specific knockout of TDP43 in macrophages obviously alleviates western diet-induced AS progression in mice.

**Conclusions:**

Transactive response DNA-binding protein∼43 kDa exacerbates atherosclerosis progression by promoting inflammation and lipid uptake of macrophages, suggesting TDP43 as a potential target for developing atherosclerotic drug.

## Introduction

Atherosclerosis (AS), which is a chronic immune-inflammatory disease characterized by cholesterol overloaded-macrophages accumulation and plaque formation in blood vessels, is the major cause of cardiovascular disease, such as coronary artery disease (CAD), and stroke (Go et al., 2013; Writing Group Members et al., 2016). Macrophages, as the primary immune cells present in plaque, have been reported to play essential roles in the development of AS through formation of foam cells and production of inflammatory factors (Hansson and Hermansson, 2011; Moore et al., 2013). Nowadays, the therapeutics of AS remain undesirable. Thus, illuminating the underlying mechanism of macrophage-mediated foam cell formation and inflammatory responses in atherosclerosis will deeper our understanding of disease and provide potential targets for developing novel drugs for AS therapy.

Transactive response DNA-binding protein∼43 kDa (TDP43), as a nuclear deoxyribonucleic acid (DNA)/RNA binding protein, acts multiple roles on RNA metabolism, such as transport of RNA, transcription, translation (Ling et al., 2013; Coyne et al., 2017). Nowadays, mis-localization and aggregation of TDP43 in the cytoplasm has been considered as an independent driver of neurodegenerative diseases, including Alzheimer’s disease, frontotemporal lobar degeneration, amyotrophic lateral sclerosis (Uryu et al., 2008; Yarchoan et al., 2012; Nag et al., 2015; Yu L. et al., 2020). In mechanism, mis-localization of TDP43 in the cytoplasm is found to destroy mitochondria membrane potential, disturb metal ion homeostasis and chromatin remodeling, and induce oxidative stress (Wang et al., 2016; Xia et al., 2016; Berson et al., 2017). Moreover, TDP43 is demonstrated to locate to mitochondria and trigger mitochondrial DNA release to activate cyclic GMP-AMP synthase (cGAS)/stimulator of type 1 interferon gene (STING) signaling, resulting in induction of inflammatory responses and type I IFN production in amyotrophic lateral sclerosis (Yu C.H. et al., 2020; Kumar and Julien, 2021). Recently, TDP43 pathology has been reported to be associated with brain arteriolosclerosis (Katsumata et al., 2018). However, whether TDP43 is involved in atherosclerosis development, especially in macrophages-mediated-foam cell formation and inflammatory responses is still unclear.

In the present study, we explored the roles of TDP43 on macrophage-mediated inflammatory responses and lipid uptake during AS progression *in vitro* and *in vivo*. Our study for the first time demonstrates that TDP43 promotes oxidized low-density lipoprotein (oxLDL)-induced mitochondrial DNA release to trigger inflammation in macrophages. Besides, TDP43 strengthens lipid uptake of macrophages through regulating β-catenin and peroxisome proliferator-activated receptor gamma (PPAR-γ) complex to promote Cluster of Differentiation 36 Receptor (CD36) transcription. Moreover, specific knockout of TDP43 in macrophages obviously alleviates western diet-induced AS progression in mice. Our study suggests TDP43 as a potential target for developing atherosclerotic drug.

## Materials and Methods

### Cell Culture and Reagents

THP-1 macrophages, purchased from ATCC (United States), were cultured in complete RPMI-1640 (Gibco, United States) medium containing 10% fetal bovine serum (Gibco, United States). The THP-1 cells were stimulated with phorbol ester (PMA) (100 nM, Sigma, United States) to differentiate macrophage-like sticky cells for later experiments. Peritoneal macrophages were obtained 3 days after intraperitoneal injection of 2 ml of 3% thioglycolate broth, 2 ml of 3% proteose peptone or 25 μg/ml of concanavalin A. Macrophages were isolated by peritoneal lavage with 10 ml ice-cold PBS/EDTA and cultured in high glucose (25 mM) DMEM supplemented with 10% LPDS (Sigma-Aldrich, S5394), 1% penicillin-streptomycin for 2 h to adhere to the non-treated culture dish, as previously reported (Schon-Hegrad and Holt, 1981). Human oxLDL was purchased from Guangzhou Yiyuan (China). Cyclosporine A (CsA) (the inhibitor of mitochondrial permeability transition pore, 12.5 μM), XAV939 (β-catenin signaling inhibitor, 5 nM), Ethidium bromide (EtBr) (50 ng/ml), and pyrrolidine dithiocarbamate (PDTC) (an inhibitor of the nuclear factor-kappa B, 10 μM) were purchased from Sigma biotech (United States). SC75741 (another inhibitor of the nuclear factor-kappa B, 1 μM), sulfo-N-succinimidyl oleate (SSO) (an inhibitor of CD36, 50 μM), and T0070907 (PPAR-γ inhibitor, 2 nM) were obtained from Selleck Chemicals (United States).

### Real Time-PCR (RT-PCR)

Real Time-polymerase chain reaction (RT-PCR) was performed as previously described (Huangfu et al., 2020). Briefly, after treatment, total RNA was extracted using the RNA elution kit (Tiangen, China). Subsequently, cDNA was generated using the PrimeScript 1st strand cDNA synthesis kit (Takara, Japan). The SYBR premix Ex Taq II (Takara, Japan) master mix was used to perform the RT-PCR reactions, and the amplicons were detected using the ABI PRISM 7500 Detection System (United States). Expression of mRNAs level was normalized to GAPDH. Primers used for RT-PCR were as follows: TDP43, F: GGGTAACCGAAGATGAGAACG, R: CTGGGCTGTAACCGT GGAG; IL6, F: ACTCACCTCTTCAGAACGAATTG, R: CCA TCTTTGGAAGGTTCAGGTTG; tumor necrosis factor-alpha (TNF-α), F: CCTCTCTCTAATCAGCCCTCTG, R: GAGGAC CTGGGAGTAGATGAG; MT-ND1 (mitochondrial ND1 gene), F: CTCTTCGTCTGATCCGTCCT, R: TGAGGTTGCGGTCT GTTAGT; 18SSRNA (18S ribosomal RNA), F: GTAACCC GTTGAACCCCATT, R: CCATCCAATCGGTAGTAGCG; CD 36, F: GGCTGTGACCGGAACTGTG, R: AGGTCTCCAACT GGCATTAGAA; macrophage scavenger receptor 1 (MSR1), F: GCAGTGGGATCACTTTCACAA, R: AGCTGTCATTGA GCGAGCATC; OLR1, F: TTGCCTGGGATTAGTAGTGACC, R: GCTTGCTCTTGTGTTAGGAGGT; ABCA1, F: ACCCAC CCTATGAACAACATGA, R: GAGTCGGGTAACGGAAACA GG; ABCG1, F: ATTCAGGGACCTTTCCTATTCGG, R: CT CACCACTATTGAACTTCCCG; GAPDH, F: TGATGGGTGT GAACCACGAG, R: AGTGATGGCATGGACTGTGG.

### Western Blot

Western blot was performed as previously described (Huangfu et al., 2018). Briefly, cells were collected and lysed using cell lysis buffer (CST, United States). After protein quantification with a BCA kit (Beyotime, CHINA), 30 μg of protein was loaded into a SDS gel, transferred onto PVDF membranes (Millipore, United States), and then incubated with primary antibodies and secondary antibodies. Finally, the protein bands were detected using an ECL kit (Pierce, United States). The primary antibodies used were as follows: TDP43 (1:1000, Abcam, United States), GAPDH (1:3000, Abcam, United States), phosphorylated p38 (p-p38, 1:1000, Abcam, United States), p38 (1:1000, Abcam, United States), p-JNK (1:1000, Abcam, United States), JNK (1:1000, Abcam, United States), p-ERK (1:1000, Abcam, United States), ERK (1:1000, Abcam, United States), p-p65 (1:1000, Abcam, United States), p65 (1:1000, Abcam, United States), flag (1:1000, Proteintech, China), CD36 (1:1000, Abcam, China), OLR1 (1:1000, Abcam, China), MSR1 (1:1000, Millipore, China), ABCA1 (1:1000, Abcam, China), ABCG1 (1:1000, Abcam, China), PPAR-γ (1:1000, Abcam, China), and β-catenin (1:1000, Abcam, China).

### Immunofluorescence

Immunofluorescence was performed as previously described (Huangfu et al., 2018). In short, after treatments, cells were fixed with 4% paraformaldehyde (Sigma, United States), permeabilized with 0.1% Triton X-100 (Sangon, China) and blocked in PBS containing 5% BSA for 1 h at room temperature. Cells were stained with anti-TDP43, anti-CD206, anti-ly6C, or anti-p-p65 (1:100) in PBS at 4°C overnight. After three washes with PBS, the cells were further incubated with second antibodies anti-Rat IgG or anti-mouse IgG (Abcam, 1:500) in PBS for 1 h at room temperature. Nuclei were stained for 5 min at room temperature in PBS containing DAPI (0.5 μg/ml, Cell signaling). Images were acquired by a Carl Zeiss LSM 510 confocal. Cell fluorescence intensity was quantified with Image J software (United States) in at least more than 50 cells from five random images, and relative fluorescence intensity was calculated as the targeted fluorescence intensity relative to the DAPI fluorescence intensity.

### Human PBMC Isolation

Human peripheral blood sample from health volunteers or patients with CAD were obtained from the Ningbo First Hospital, which was allowed by the ethics committee of Ningbo First Hospital. Human primary macrophages were induced from monocytes, which were separated from human peripheral blood with human lymphocyte separation medium (Sigma).

### Plasmids, Small Interfering RNAs (siRNAs) and Lentiviruses

Small interfering RNAs (siRNAs) of TDP43 or cGAS were synthetized and purchased from Shanghai GenePharma Co., Ltd. (China). pCMV3-N-FLAG-TDP43 plasmid was obtained from Sino Biological Co., Ltd. (China). siRNAs and plasmid were transfected with jetPRIME transfection reagent (polyplus, FRANCE), according to the manufacture’s instruction. In short, cells were cultured in six-well plates with completed medium. A total of 2 μL of siRNA and 4 μL of jetPRIME transfection reagent were mixed, incubated for 10 min, and added to each well. After 24 h, the cells were collected for the following experiments. Lentivirus-expressing flag-tagged TDP43 (Lenti-TDP43) and control lentivirus (Lenti-Control) were synthetized and purchased from RIBOBIO (China).

### Protein Immunoprecipitation (co-IP)

co-IP was performed as previously described (Huangfu et al., 2020). Briefly, after treatment, cells were collected and lysed with cell lysis buffer (CST, United States). Then, cell lysates were pre-cleared by incubation with 20 μL of protein A/G agarose (Santa Cruz, United States) for 1 h and incubated with anti-Flag/TDP43/β-Catenin antibodies at 4°C with rotation overnight. After centrifugation, the supernatant was incubated with 80 μL of protein A/G agarose at 4°C with rotation for 4 h. The agarose beads were washed three times with cold PBS, followed by elution with 40 μL of protein lysis buffer. Then, 10 μL of 5× loading buffer (Beyotime, Shanghai, China) was added, and the mixture was boiled for 5 min and subjected to western blot.

### Enzyme-Linked Immunosorbent Assay (ELISA)

After treatment, the secretory TNF-α or IL6 proteins in the supernatant of cellular culture were measured using the Human TNF-α Enzyme-Linked Immunosorbent Assay (ELISA) Kit (Abcam, United States) or the Human interleukin-6 (IL-6) ELISA Kit (Abcam, United States), according to the manufacturer’s instructions.

### Lipid Uptake Analysis

After treatments, the cells were treated with dil-oxLDL (10 ng/ml, Yiyuan, China) for 30 min. Next, the cells were washed twice by ice-cold PBS and observed using the Zeiss Confocal Microscope Imaging System (Carl Zeiss, Germany).

### Chromatin Immunoprecipitation (ChIP) Assays

Chromatin immunoprecipitation (ChIP) assays were performed with the SimpleChIP^®^ Enzymatic Chromatin IP Kit (Cell Signaling Technology, United States) as previously described (Huangfu et al., 2018). In brief, 2 × 10^7^ macrophages were fixed with 1% formaldehyde for 10 min at 37°C. Next, chromatin was sheared with 5 μL of nuclease at 37°C for 20 min, yielding DNA fragments of 200–800 bp. Following pre-clearing with 20 μL of protein A/G agarose at 4°C for 1 h, the samples were incubated with 2 μg of anti-p65/β-catenin/PPAR-γ/TDP43 antibody or control IgG antibody at 4°C with rotation overnight. Complexes including input were incubated in 5 M NaCl at 65°C for 6 h to reverse crosslinks, resuspended in proteinase K solution at 45°C for 1 h, and then purified using a DNA purification kit (Beyotime, China). The purified DNA was subjected to polymerase chain reaction (PCR) analyses. The primers used for PCR detection were as follows: CD36 promoter binding sites for β-catenin and PPAR-γ complex, F: GCGGTACGCAGAGTCGACTC, R: AGGATACGGGTCTTGCACAG; IL-6 promoter binding sites for p65, F: AGACCAGTGATTTTCACCAGG, R: TGGCATGAGCTGAGGGTTATTGC; TNF-α promoter binding sites for p65, F: CCTCTCCTTTGGCCATTCCAAGC, R: CATGCCCCTCAAAACCTATTGCC.

### Mitochondrial DNA-Depleted Cells (ρ^0^)

THP-1 cells were cultured in a selective medium containing ethidium (100 ng/mL), sodium pyruvate (100 μg/mL) and urdine (50 μg/mL) for 3 weeks and later rested for an additional 2 weeks in the presence of uridine aiming to get mtDNA-depleted cell lines which had been described previously (Hashiguchi and Zhang-Akiyama, 2009).

### Animal Experiments

Macrophage specific TDP43 knockout ApoE^–/–^ mice: ApoE^–/–^ mice (purchased from Model Animal Research Center of Nanjing University) with loxP sites flanking exon 2 of the TDP43 gene (TDP43 fl/fl) were crossed with CSF1R-Cre transgenic mice to generate mice with a macrophage-specific deletion of TDP43 (TDP43 M-KO, −/−). All mice were maintained in an animal facility at an ambient temperature of 20–24°C with 40–70% relative humidity under a 12:12 h light-dark cycle. All mouse experiments were conducted after approval by the ethics committee of Ningbo First Hospital. Mice (8 weeks old, including TDP43 fl/fl mice and TDP43 M-KO mice) were fed a high fat diet (16.9% fat, 1.3% cholesterol, 21.1% crude protein, and 46.5% carbohydrates) for 12 weeks to construct a mouse model of atherosclerosis. Once the model of atherosclerosis was established (20 weeks old), mice were sacrificed by CO_2_ asphyxiation. Aorta from the ascending aorta to the arteria iliaca communis was isolated and stained using oil red (Jiancheng, China). Atherosclerotic lesion sizes were assessed using Image Pro Plus 6.0 and expressed as the percentage of plaque area relative to the total intimal area.

### Statistics Analysis

Data throughout the paper are expressed as mean ± SD. Statistical differences were calculated using unpaired two-tailed Student’s *t* test or one-way ANOVA with Bonferroni correction for multiple comparisons. A probability of *P* < 0.05 was considered statistically significant. Ns, not significant.

## Results

### TDP43 Expression Is Upregulated in oxLDL-Treated Macrophages and Peripheral Blood Mononuclear Cells From Patients With Coronary Artery Disease

**FIGURE 1:**
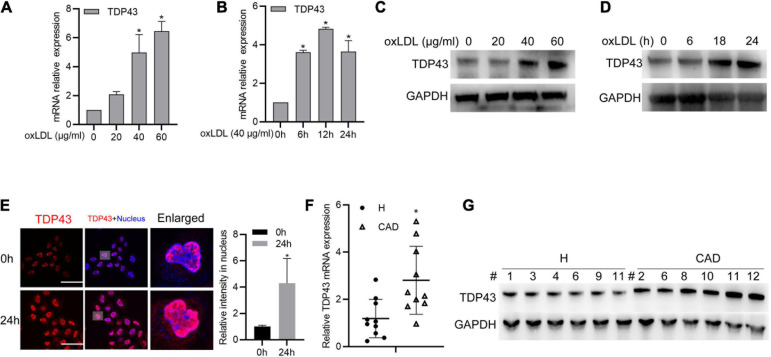
Transactive response DNA-binding protein∼43 kDa (TDP43) expression is upregulated in oxLDL-treated macrophages and peripheral blood mononuclear cells from patients with CAD. **(A)** qPCR detection of TDP43 mRNA in macrophages treated with the indicated concentrations of oxLDL for 24 h. Data are represented as means ± SD (*n* = 3; *P < 0.05 vs. 0 μg/ml oxLDL stimulated group). **(B)** qPCR detection of TDP43 mRNA in macrophages treated with 40 μg/mL oxLDL for the indicated time points. Data are represented as means ± SD (*n* = 3; *P < 0.05 vs. 40 μg/ml oxLDL stimulated for 0 h group). **(C)** Western blot analysis of TDP43 expression in macrophages treated with the indicated concentrations of oxLDL for 24 h. GAPDH was used as a loading control. **(D)** Western blot analysis of TDP43 expression in macrophages treated with 40 μg/mL oxLDL for the indicated time points. **(E)** Immunofluorescence analysis of TDP43 cellular distribution in macrophages treated with or without 40 μg/mL oxLDL for 24 h. Data are represented as means ± SD (*n* = 50; *P < 0.05 vs. 40 μg/ml oxLDL stimulated for 0 h group). Scale bar = 100 μm. **(F)** qPCR detection of TDP43 mRNA in PBMCs isolated from health volunteers (H) or patients with (CAD) Data are represented as means ± SD (*n* = 10; *represents *P* < 0.05 vs. H group) **(G)** Western blot analysis of TDP43 expression in PBMCs isolated from health volunteers or patients with CAD. GAPDH was used as a loading control.

To explore the potential role of TDP43 during AS development, we firstly detected TDP43 mRNA/protein expressions in macrophages treatment with oxidized LDL (oxLDL), and found that oxLDL stimulation evidently promoted TDP43 mRNA/Protein expressions in a dose/time dependent manner (Figures 1A–D). Furthermore, we found that TDP43 protein exclusively distributed in nucleus of macrophages under normal condition, while oxLDL stimulation induced partial TDP43 protein translocated into cytoplasm (Figure 1E). This mis-location of TDP43 protein suggests that TDP43 may be involved in the pathogenesis of AS. Subsequently, we further examined the TDP43 expression level in peripheral blood mononuclear cells (PBMCs) from health volunteers or patients with CAD. As expected, TDP43 mRNA/protein expression levels are indeed higher in PBMCs from patients with CAD, compared to those in health volunteers (Figures 1F,G). In addition, consistent with the results of Figure 1C, the protein expression levels of TDP43 are also significantly increased in oxLDL-stimulated PBMCs (Supplementary Figure 1A). Collectively, these results implicate that TDP43 expression level is relatively higher in oxLDL-treated macrophages or peripheral blood mononuclear cells from patients with CAD.

### TDP43 Promotes oxLDL-Induced Inflammation and Foam Cell Formation in Macrophages

**FIGURE 2:**
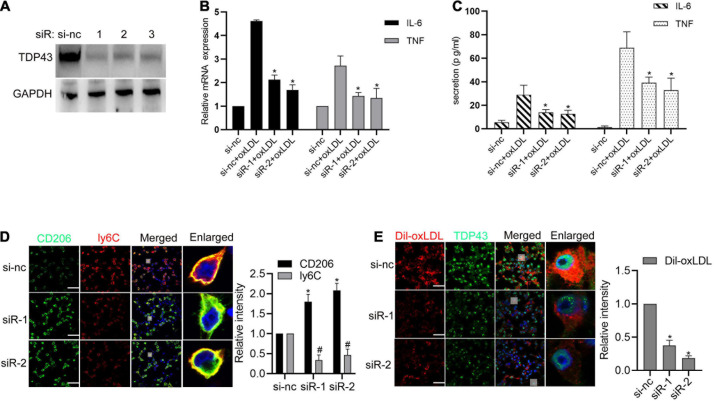
Transactive response DNA-binding protein∼43 kDa (TDP43) promotes oxLDL-induced inflammation and foam cell formation in macrophages. **(A)** Western blot analysis of TDP43 expression in macrophages transfected with TDP43 specific siRNA (−1, −2, −3) or a non-specific negative control siRNA (si-nc) for 24 h. GAPDH was used as a loading control. **(B)** qPCR detection of IL-6 and TNF mRNAs in macrophages transfected with TDP43 specific siRNA (siR-1, siR-2) or si-nc for 24 h and later incubated with 40 μg/mL oxLDL for 24 h. Data are represented as means ± SD (*n* = 3; *P < 0.05 vs. si-nc + oxLDL group). **(C)** ELISA detection of IL-6 and TNF expressions in the culture supernatants of macrophages transfected with TDP43 specific siRNA (siR-1, siR-2) or si-nc for 24 h and later incubated with 40 μg/mL oxLDL for 24 h. Data are represented as means ± SD (*n* = 3; *P < 0.05 vs. si-nc + oxLDL group). **(D)** Immunofluorescence analysis of CD206 and ly6C expression in macrophages transfected with TDP43 specific siRNA (−1, −2) or si-nc for 24 h. Data are represented as means ± SD (*n* = 50; * or ^#^*P* < 0.05 vs. si-nc group). Scale bar = 100 μm. **(E)** Immunofluorescence analysis of Dil-oxLDL uptake of macrophages transfected with TDP43 specific siRNA (−1, −2) or si-nc for 24 h. Data are represented as means ± SD (*n* = 50; * or ^#^*P* < 0.05 vs. si-nc group). Scale bar = 100 μm.

Given that macrophages-related inflammation and lipid accumulation play essential roles in AS (Poznyak et al., 2020; Gencer et al., 2021), we further explored whether TDP43 is involved in these biological processes. To verify this hypothesis, TDP43 specific siRNAs were synthesized. As shown in Figure 2A, all of the three TDP43 siRNAs could significantly reduce TDP43 protein expression in macrophages and we chose siRNA#1 and siRNA#2 used for the following experiments. Knockdown of TDP43 in macrophages or PBMCs evidently suppressed oxLDL-induced pro-inflammatory factors, IL-6 and TNF-α mRNA transcription (Figure 2B and Supplementary Figures 1B,C) and secretion (Figure 2C and Supplementary Figure 1D). Considering that macrophages were generally induced to polarize into M1 phenotype which produces pro-inflammatory factors to promote AS development, while, M2 phenotype produces anti-inflammatory factors (Liu et al., 2020; Peng et al., 2020), we further explored the effect of TDP43 in macrophage polarization. As shown in Figure 2D and Supplementary Figure 2, knockdown of TDP43 evidently increased the M2 marker, CD206 expression in macrophages, and decreased the M1 marker, Ly6C expression. Moreover, knockdown of TDP43 significantly inhibited lipid uptake of macrophages (Figure 2E). Overall, these results indicate that TDP43 promotes oxLDL-induced inflammation and foam cell formation in macrophages.

### TDP43 Promotes Activation of NF-κB Signaling to Increase Inflammatory Factor Expression in Macrophages

**FIGURE 3:**
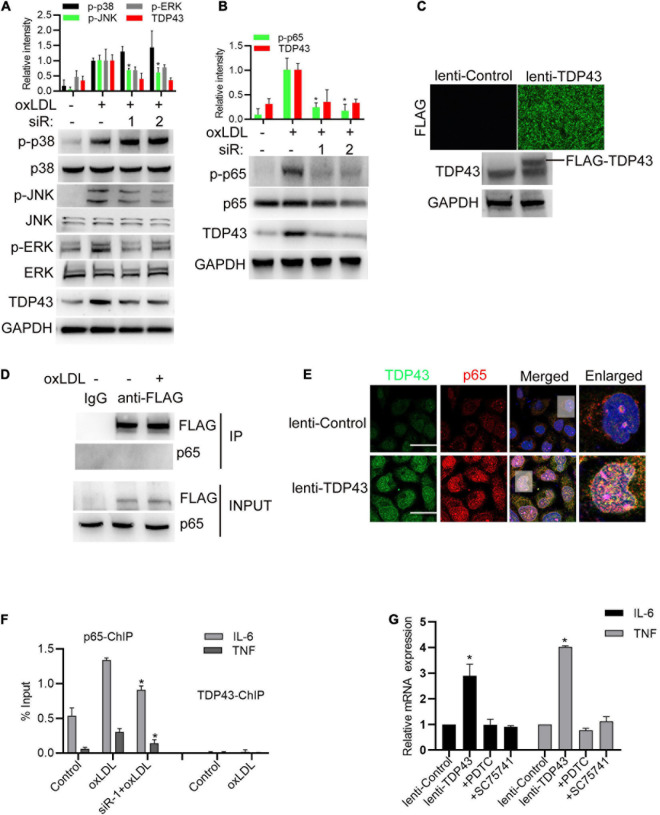
Transactive response DNA-binding protein∼43 kDa (TDP43) promotes activation of NF-κB signaling to increase inflammatory factor expression in macrophages. **(A,B)** Western blot analysis of MAPKs and NF-κB signaling in macrophages transfected with TDP43 specific siRNA (−1, −2) or si-nc for 24 h and later incubated with 40 μg/mL oxLDL for 24 h. Data are represented as means ± SD (*n* = 3; *P < 0.05 vs. oxLDL stimulated group). **(C)** Immunofluorescence analysis of FLAG-tagged TDP43 expression in macrophages infected with lenti-control or lenti-TDP43 for 48 h (upper panel). Western blot analysis of TDP43 in macrophages infected with lenti-control or lenti-TDP43 for 48 h with GAPDH as a loading control (down panel). **(D)** Co-IP detection of the interaction between FLAG-tagged TDP43 and p65 in macrophages infected with lenti-control or lenti-TDP43 for 24 h and later incubated with or without 40 μg/mL oxLDL for 24 h by using FLAG antibody. **(E)** Immunofluorescence analysis of TDP43 and phosphorylated p65 (p-p65) in macrophages infected with lenti-control or lenti-TDP43 for 48 h. Scale bar = 40 μm. **(F)** ChIP analysis of the binding of p65 or TDP43 to IL-6 or TNF promoter in macrophages transfected with TDP43 specific siRNA (-1) or si-nc for 24 h and later incubated with 40 μg/mL oxLDL for 24 h. Data are represented as means ± SD (*n* = 3; *P < 0.05 vs. oxLDL stimulated group). **(G)** q-PCR detection of IL-6 and TNF mRNA expressions in macrophages incubated with PDTC or SC75741 (two NF-KB signaling inhibitors with different targets) for 1 h and later infected with lenti-control or lenti-TDP43 for 48 h Data are represented as means ± SD (*n* = 3; *P < 0.05 vs. lenti-control group).

To gain further insights into the molecular mechanism of TDP43 promoting inflammatory response, we examined whether TDP43 affects the canonical inflammation-related mitogen-activated protein kinases (MAPK) signalings, including extracellular signaling-regulated kinase (ERK1/2), c-Jun N-terminal kinase (JNK) and p38 MAPK, and NF-κB (nuclear factor kappa-light-chain-enhancer of activated B cells) signaling, which are activated to promote inflammation and play important roles in the pathogenesis of AS (Bryk et al., 2014; Shahi et al., 2019). As shown in Figure 3A, TDP43 knockdown weakly enhanced oxLDL-induced p38 phosphorylation, but decreased oxLDL-induced JNK1/2 and ERK1/2 phosphorylation in macrophages. Whereas, TDP43 knockdown prominently reduced oxLDL-induced p65 phosphorylation in macrophages (Figure 3B), suggesting that NF-κB signaling may be mediated TDP43-induced inflammation.

Subsequently, lentivirus expressing FLAG-tagged TDP43 was packaged and could efficiently infect macrophages (Figure 3C). Through protein immunoprecipitation (co-IP) analysis, we found that TDP43 did not directly interact with p65 (Figure 3D). But, overexpression of TDP43 prominently induced p65 phosphorylation, and obviously co-localized with p65 in nucleus of macrophages (Figure 3E). Based on these findings, we hypothesized TDP43 may promote IL-6 and TNF-α transcription through enhancing p65 phosphorylation. Through chromosome immunoprecipitation (ChIP) analysis, TDP43 did not directly bind to the promoter region of IL-6 and TNF-α, whereas TDP43 knockdown significantly reduced p65 binding to the promoter of IL-6 and TNF-α (Figure 3F). Moreover, to further confirm our hypothesis, two NF-κB specific inhibitors (PDTC and SC75741) were used to pre-block p65 activation before TDP43 overexpression in macrophages and we found that inhibition of NF-κB signaling nearly canceled TDP43 overexpression-induced IL-6/TNF-α mRNA transcription (Figure 3G). Overall, these results indicate that TDP43 promotes activation of NF-κB signaling to increase inflammatory factor expression in macrophages.

### TDP43 Mediates oxLDL-Induced mtDNA Release to Activate Inflammation

**FIGURE 4:**
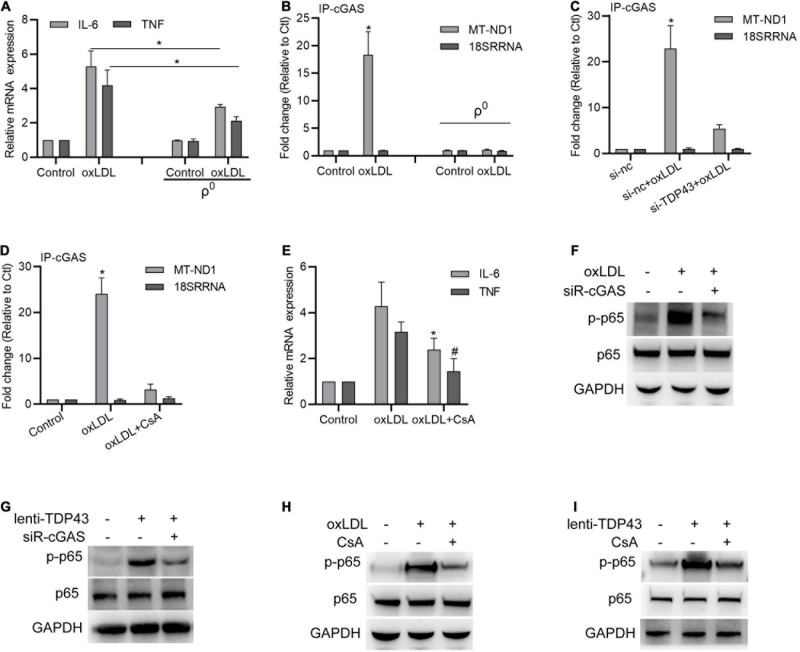
Transactive response DNA-binding protein∼43 kDa (TDP43) mediates oxLDL-induced mtDNA release to activate inflammation. **(A)** qPCR detection of IL-6 and TNF mRNA in macrophages or mtDNA depleted macrophages (ρ^0^) treated with 40 μg/mL oxLDL or not for 24 h. Data are represented as means ± SD (*n* = 3; *P < 0.05). **(B)** qPCR detection of MT-ND1 (mitochondrial DNA) and 18SSRNA (nuclear DNA) in cGAS immunoprecipitants from macrophages or mtDNA depleted macrophages (ρ^0^) treated with 40 μg/mL oxLDL or not for 24 h. Data are represented as means ± SD (*n* = 3; *P < 0.05 vs. control group). **(C)** qPCR detection of MT-ND1 (mitochondrial DNA) and 18SSRNA (nuclear DNA) in cGAS immunoprecipitants from macrophages transfected with si-TDP43 or si-nc for 24 h and later incubated with 40 μg/mL oxLDL for 24 h. Data are represented as means ± SD (*n* = 3; *P < 0.05 vs. si-nc group). **(D)** qPCR detection of MT-ND1 (mitochondrial DNA) and 18SSRNA (nuclear DNA) in cGAS immunoprecipitants from macrophages with or without 12.5 μM CsA (inhibition of mtDNA leakage) incubation and later incubated with 40 μg/mL oxLDL for 24 h. Data are represented as means ± SD (*n* = 3; *P < 0.05 vs. control group). **(E)** qPCR detection of IL-6 and TNF mRNA in macrophages with or without CsA incubation and later incubated with 40 μg/mL oxLDL for 24 h. Data are represented as means ± SD (*n* = 3; * or ^#^*P* < 0.05 vs. oxLDL group). **(F)** Western blot analysis of p65 and p-p65 protein expressions in macrophages transfected with si-cGAS for 24 h and later incubated with 40 μg/mL oxLDL for 24 h. **(G)** Western blot analysis of p65 and p-p65 protein expressions in macrophages transfected with si-cGAS for 24 h and later infected with lenti-control or lenti-TDP43 for 48 h. **(H)** Western blot analysis of p65 and p-p65 protein expressions in macrophages with or without CsA incubation and later incubated with 40 μg/mL oxLDL for 24 h. **(I)** Western blot analysis of p65 and p-p65 protein expressions in macrophages with or without CsA incubation and later infected with lenti-control or lenti-TDP43 for 48 h.

Considering that TDP43 has recently been reported to trigger inflammation by inducing mitochondrial DNA (mtDNA) release to activate cGAS (cyclic guanosine monophosphate-AMP synthase)/STING (The stimulator of interferon genes) in amyotrophic lateral sclerosis (Yu C.H. et al., 2020), we hypothesized whether TDP43 activates p65 phosphorylation in macrophages under oxLDL stimulation is also associated with induction of mtDNA release. To test our hypothesis, we firstly constructed mtDNA depleted macrophages (ρ^0^ cell lines) by culturing them with EtBr (Hashiguchi and Zhang-Akiyama, 2009). As expected, mtDNA depletion in macrophages indeed partly reversed oxLDL-induced IL-6 and TNF-α mRNA transcription (Figure 4A). Next, we performed R-IP using cGAS specific antibodies and found oxLDL treatment prominently strengthens cGAS binding to mtDNA (MT-ND1) but not nuclear DNA (18SRRNA), and depletion of mtDNA abolished cGAS binding to mtDNA (Figure 4B), suggesting that oxLDL stimulation indeed promotes mtDNA release to activate cGAS. Moreover, either knockdown of TDP43 or inhibition of mtDNA leakage with CsA canceled oxLDL-induced cGAS binding to mtDNA (Figures 4C,D). Besides, mtDNA leakage inhibition by using CsA also suppressed oxLDL-induced IL-6 and TNF-α mRNA expression (Figure 4E), which indicates that mtDNA release-induced by oxLDL is associated with oxLDL-mediated inflammation. To further confirm the role of cGAS during oxLDL-induced inflammation, cGAS expression was downregulated by siRNA transfection. As shown in Figures 4F,G, knockdown of cGAS evidently suppressed oxLDL stimulation or TDP43 overexpression-induced p65 phosphorylation in macrophages. In addition, inhibition of mtDNA leakage also partly reversed oxLDL stimulation or TDP43 overexpression-induced p65 phosphorylation (Figures 4H,I). Token together, these results indicate that TDP43 mediates oxLDL-induced mtDNA release to activate inflammation.

### TDP43 Strengthens Lipid Uptake of Macrophages Through Promoting CD36 Expression

**FIGURE 5:**
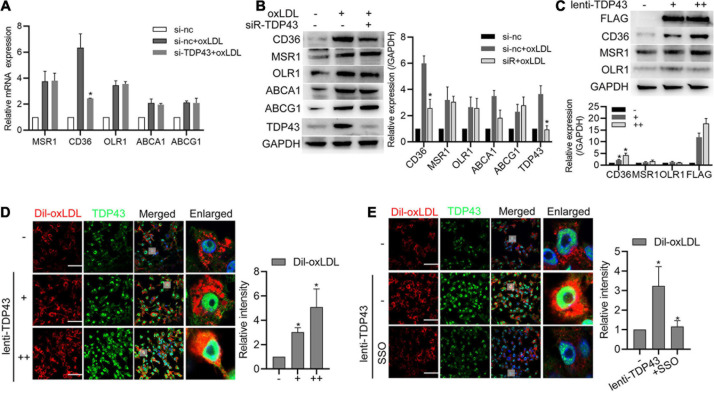
Transactive response DNA-binding protein∼43 kDa (TDP43) strengthens lipid uptake of macrophages through promoting Cluster of Differentiation 36 Receptor (CD36) expression. **(A)** qPCR detection of lipid uptake and export related genes in macrophages transfected with si-TDP43 or si-nc for 24 h and later incubated with 40 μg/mL oxLDL for 24 h. Data are represented as means ± SD (*n* = 3; *P < 0.05 vs. si-nc + oxLDL group). **(B)** Western blot analysis of lipid uptake and export related proteins in macrophages transfected with si-TDP43 or si-nc for 24 h and later incubated with 40 μg/mL oxLDL for 24 h. Data are represented as means ± SD (*n* = 3; *P < 0.05 vs. si-nc + oxLDL group). **(C)** Western blot analysis of CD36, MSR1, and OLR1 protein expressions in macrophages infected with lenti-control or lenti-TDP43 for 48 h. Data are represented as means ± SD [*n* = 3; *P < 0.05 vs. lenti-TDP43 (–) group]. **(D)** Immunofluorescence analysis of Dil-oxLDL uptake of macrophages infected with lenti-control or lenti-TDP43 for 48 h. Data are represented as means ± SD [*n* = 50; *P < 0.05 vs. lenti-TDP43 (–) group]. Scale bar = 100 μm. **(E)** Immunofluorescence analysis of Dil-oxLDL uptake of macrophages incubated with or without CD36 specific inhibitor (sulfo-N-succinimidyl oleate, SSO, 50 μM) and later infected with lenti-control or lenti-TDP43 for 48 h. Data are represented as means ± SD (*n* = 50; *P < 0.05 vs. lenti-TDP43 group). Scale bar = 100 μm.

To further investigate the underlying mechanism of TDP43 regulating macrophage lipid uptake, we firstly examined the effect of TDP43 on scavenger receptors, such as CD36, MSR1, and OLR1 (also known as lectine like oxidized low density lipoprotein receptor-1, LOX1), which are responsible for lipid uptake in macrophages (Crucet et al., 2013; Tian et al., 2020), and reverse transporters of cholesterol from macrophages, such as ATP−binding cassette (ABC) transporters ABCA1 and ABCG1 (Feingold, 2000; Cai et al., 2020). As shown in Figures 5A,B, oxLDL stimulation significantly increased CD36, MSR1, OLR1, ABCA1, and ABCG1 mRNA/protein expressions, whereas TDP43 knockdown specifically suppressed CD36 mRNA/protein expressions. Consistently, overexpression of TDP43 evidently promoted CD36 protein expression and lipid uptake of macrophages (Figures 5C,D). Moreover, CD36 inhibitor (SSO) was used to explore whether TDP43 promoting lipid uptake of macrophages depends on CD36. The result of Figure 5E showed that inhibition of CD36 prominently inhibited TDP43 overexpression-induced lipid uptake. Token together, these results indicate that TDP43 strengthens lipid uptake of macrophages through promoting CD36 expression.

### TDP43 Strengthens CD36 Transcription by Regulating β-Catenin and PPAR-γ Complex Formation

**FIGURE 6:**
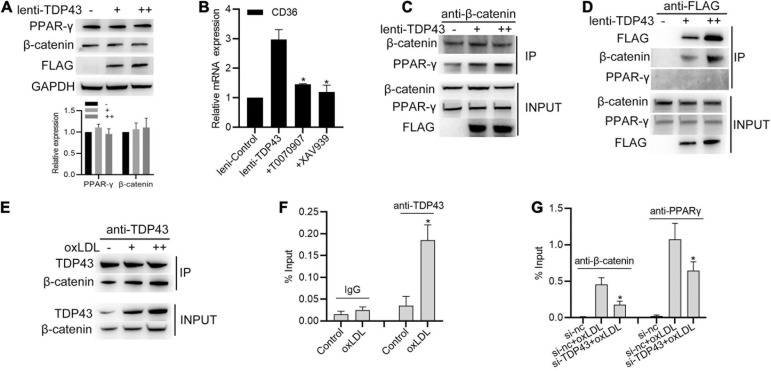
Transactive response DNA-binding protein∼43 kDa (TDP43) strengthens CD36 transcription by regulating β-catenin and PPAR-γ complex formation. **(A)** Western blot analysis of PPAR-γ and β-catenin protein expressions in macrophages infected with lenti-control or lenti-TDP43 for 48h. GAPDH was used as a loading control. **(B)** qPCR detection of CD36 mRNA in macrophages incubated with T0070907 (PPAR-γ inhibitor, 2 nM) or XAV939 (β-catenin signaling inhibitor, 5 nM) and later infected with lenti-control or lenti-TDP43 for 48 h. Data are represented as means ± SD (*n* = 3; *P < 0.05 vs. lenti-TDP43 group). **(C)** Co-IP analysis of the interaction between β-catenin and PPAR-γ in macrophages infected with lenti-control or lenti-TDP43 for 48 h. **(D)** Co-IP analysis of interactions among flag-tagged TDP43, β-catenin and PPAR-γ in macrophages infected with lenti-control or lenti-TDP43 for 48h by using FLAG antibody to specific precipitate FLAG-tagged TDP43 protein complex. Scale bar = 100 μm. **(E)** Co-IP analysis of the interaction between β-catenin and TDP43 in macrophages infected with or without oxLDL (40 μg/mL: +; 60 μg/mL: ++) for 24 h using TDP43 antibody to specific precipitate TDP43 protein complex. Scale bar = 100 μm. **(F)** ChIP analysis of the binding of TDP43 (IgG used as negative control) to CD36 promoter in macrophages with 40 μg/mL oxLDL incubation for 24 h. Data are represented as means ± SD (*n* = 3; *P < 0.05 vs. control group). **(G)** ChIP analysis of the binding of PPAR-γ or β-catenin to CD36 promoter in macrophages transfected with si-TDP43 or si-nc for 24 h and later incubated with 40 μg/mL oxLDL for 24 h. Data are represented as means ± SD (*n* = 3; *P < 0.05 vs. si-nc + oxLDL group).

Previous studies reported that oxLDL stimulation increased β-catenin interacting with PPAR-γ to form the β-catenin-PPAR-γ complex, which binds to the DR-1 motif sequence of CD36 promoter to promote CD36 transcription (Tontonoz et al., 1998; Wang et al., 2015). Thus, we hypothesized whether TDP43 promotes CD36 transcription through regulating β-catenin or PPAR-γ. To our surprise, overexpression of TDP43 had no obvious effect on β-catenin or PPAR-γ protein expression (Figure 6A). However, blocking PPAR-γ with T0070907 or β-catenin with XAV939 significantly suppressed TDP43 induced CD36 transcription (Figure 6B), suggesting that TDP43 promoting CD36 transcription in macrophages may be related to β-catenin and PPAR-γ. Furthermore, we found that overexpression of TDP43 promoted β-catenin interacted with PPAR-γ in macrophages (Figure 6C). Interestingly, exogenous flag-tagged TDP43 or endogenous TDP43 could specifically interacted with β-catenin, but not PPAR-γ (Figures 6D,E). Moreover, considering TDP43 as a DNA binding protein (Ling et al., 2013), we subsequently detected whether TDP43 binds to CD36 promoter. ChIP analysis showed that TDP43 did not directly bind to CD36 promoter in macrophages under normal conditions, whereas oxLDL stimulation induced TDP43 binding to CD36 promoter (Figure 6F). In addition, knockdown of TDP43 evidently decreased oxLDL induced β-catenin or PPAR-γ binding to CD36 promoter (Figure 6G). Collectively, these results indicate that TDP43 strengthens CD36 transcription by regulating β-catenin and PPAR-γ complex formation.

### Specific Knockout of TDP43 in Macrophages Alleviates AS Progression *in vivo*

**FIGURE 7:**
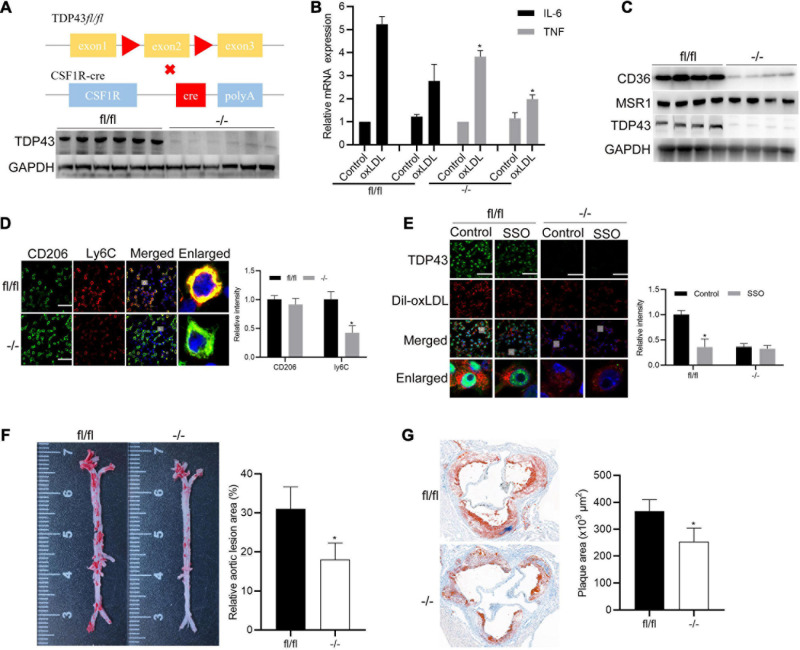
Specific knockout of TDP43 in macrophages alleviates AS progression *in vivo*. **(A)** Schematic of macrophage specific TDP43 loss-of-function mouse model (upper panel). Western blot analysis TDP43 from thioglycolate-elicited primary peritoneal macrophages extracts from control (fl/fl) or macrophage-specific TDP43 knockout mice (–/–). GAPDH was used as loading control (down panel). **(B)** qPCR detection of IL-6 and TNF mRNA expressions in thioglycolate-elicited primary peritoneal macrophages from control (fl/fl) or macrophage-specific TDP43 knockout mice (–/–) with or without oxLDL incubation for 24 h. Data are represented as means ± SD (*n* = 3; *P < 0.05 vs. oxLDL fl/fl group). **(C)** Western blot analysis of CD36 and MSR1 protein expressions in thioglycolate-elicited primary peritoneal macrophages from control (fl/fl) or macrophage-specific TDP43 knockout mice (–/–) from western diet-induced atherosclerotic model. **(D)** Immunofluorescence analysis of CD206 and Ly6C in thioglycolate-elicited primary peritoneal macrophages from control (fl/fl) or macrophage-specific TDP43 knockout mice (–/–) from western diet-induced atherosclerotic model. Data are represented as means ± SD (*n* = 50; *P < 0.05 vs. fl/fl group). Scale bar = 100 μm. **(E)** Immunofluorescence analysis of Dil-oxLDL uptake of thioglycolate-elicited primary peritoneal macrophages from control (fl/fl) or macrophage-specific TDP43 knockout mice (–/–) with or without SSO treatment. Data are represented as means ± SD (*n* = 50; *P < 0.05 vs. control fl/fl group). Scale bar = 100 μm. **(F)** Representative images and quantification of the aorta en face lesion stained with oil red O (*n* = 6 for each group). Data are represented as means ± SD (*n* = 6; *P < 0.05 vs. fl/fl group). **(G)** Representative images and quantification of the aortic root lesion area stained with oil red O (*n* = 6 for each group). Data are represented as means ± SD (*n* = 6; *P < 0.05 vs. fl/fl group).

To explore the significance of TDP43 in macrophages during AS progression *in vivo*, we generated a macrophage-specific TDP43 knockout mouse line using a typical (CSF1R promoter driven) macrophage-specific Cre-loxP system on ApoE^–/–^ background mice (Figure 7A). Thioglycolate-elicited primary peritoneal macrophages were isolated and stimulated with oxLDL. Consistent with our previous results (Figures 2B,C), TDP43 knockout in macrophage (−/−) significantly decreased oxLDL-induced IL-6 and TNF-α transcription (Figure 7B). Besides, CD36 protein expression levels were also significantly decreased in TDP43 knockout macrophages (−/−), compared to those in the control macrophages (fl/fl) (Figure 7C). Furthermore, TDP43 knockout significantly decreased ly6C (M1 related marker) expression (Figure 7D). Subsequently, we detected the lipid uptake ability of TDP43 knockout macrophages. As expected, TDP43 knockout or inhibition of CD36 by SSO treatment evidently suppressed lipid uptake of macrophages (Figure 7E), which further confirmed that TDP43 promoting lipid uptake of macrophages depends on CD36. Finally, TDP43 knockout (−/−) and control (fl/fl) mice were fed with western diet (WD) for 12 weeks to establish AS model. Consistent to our prediction, en face aorta analysis showed that the lesion area was significantly smaller in TDP43 macrophage-specific knockout mice (−/−) (Figure 7F). Quantification of the lesion areas in the aortic roots revealed significantly smaller lesions in TDP43 macrophage-specific knockout mice (−/−) than TDP43 flox mice (fl/fl) (Figure 7G). Taken together, these results indicate that specific knockout of TDP43 in macrophages alleviates AS progression *in vivo*.

## Discussion

Cardiovascular disease is still the leading cause of death all over the world, and AS is one of the major etiologies (Kuznetsova et al., 2020). Macrophage-mediated inflammatory response and lipid uptake tightly connects with atherosclerotic progression (Ferrari et al., 2020). Specifically blocking these undesirable functions in macrophages may be an attractive choice for alleviating AS and its complications. In this study, we report that TDP43, a DNA/RNA binding protein, which frequently mutates in familial amyotrophic lateral sclerosis (Kabashi et al., 2008), promotes inflammatory factor secretion and lipid uptake in macrophages. Western diet-induced AS in macrophage-specific TDP43 knockout mice on ApoE^–/–^ background showed that TDP43 knockout in macrophages has protective roles in atherosclerotic progression.

Cyclic GMP-AMP synthase, as a critical part of anti-infective immune defense, could recognize and bind to exogenous DNA or mis-localized self-DNA to provoke inflammation and interferon responses (Kerur et al., 2018; Cohen et al., 2019). Recently, cGAS has been reported to contribute to AS development through synergistic inflammatory signaling, as well as interferons, leading to macrophage M1 polarization (Lu et al., 2021). In this study, we discover the connection between cGAS and TDP43 in AS development and firstly demonstrate that TDP43 functions as the upstream regulator of cGAS-triggered inflammation in atherosclerosis through inducing mitochondrial DNA release. Besides, our study firstly reports that TDP43 promotes lipid uptake of macrophages by strengthening CD36 gene transcription through interacting with β-catenin/PPAR-γ complex. Moreover, in our study, we also find that oxLDL-stimulation induced TDP43 binding to CD36 promoter in macrophages, indicating that besides indirectly regulating CD36 gene transcription through β-catenin/PPAR-γ complex, TDP43 may also directly affect. The underlying mechanism of TDP43 regulating CD36 gene transcription needs to be further investigated in the following studies.

Previous study reports that oxLDL sequestered by CD36 induces intracellular CD36-TLR4-TLR6 heteromerization, activating NF-κB and chemokine expression, indicating lipid uptake of macrophages positively promotes inflammatory responses during AS (Stewart et al., 2010). Besides, recent study reports that LDL/CD36 signaling in macrophages links dysregulated fatty acid metabolism to oxidative stress from the mitochondria, which drives chronic inflammation during AS (Chen et al., 2019). Moreover, NF-κB signaling also positively regulates CD36 expression (Sp et al., 2018). Combined with our study and these published studies, lipid uptake and inflammatory responses regulated by TDP43 may dynamically interact in a positive-feedback manner during AS. In addition, the canonical β-catenin signaling also regulates inflammatory response (Anuja et al., 2017). Whether β-catenin signaling is also involved in TDP43-mediated inflammation in macrophages during AS development needs to be explored. By using TDP43-knockout macrophages isolated from TDP43 macrophage-specific knockout mice (−/−), we found that TDP43 knockout almost abolished Cd36 expression in macrophages, and partially suppressed ox-LDL induced inflammatory factor expression, suggesting that TDP43 may function more significant effects on inflammation than lipid accumulation during AS, which needs further investigation in the following studies.

Immune infiltration is closely associated with the progression and prognosis of atherosclerosis (Hansson and Hermansson, 2011), and up to now, 24 immune cell types have been identified within atherosclerotic tissues (Wang et al., 2021). In this study, we explored the significance of TDP43 in macrophages during AS progression using CSF1R Cre mice to generate macrophage-specific TDP43 knockout mice. However, there is some certain limitations of these macrophage-specific TDP43 knockout mice. Even if the CSF1R Cre mice strain is a good tool for fate mapping of macrophages in development and identification of macrophage regulatory genes essential for both steady status and inflammatory conditions, the specificity of CSF1R-Cre is not high and Cre mediated deletion could also be detected in dendritic cells (DCs) (Deng et al., 2010). DCs and macrophages both belong to the myeloid lineage of blood cells (Geissmann et al., 2010), and many features of DCs, such as phagocytosis, antigen capture, and production of pro-inflammatory factors, are similar to macrophages (Geissmann et al., 2010). Recently, the abundance of immune cell types within atherosclerotic tissues was analyzed, and the results indicated that macrophages and DCs significantly increased in the atherosclerotic tissues, and the expression of macrophages was closely related to the level of the expression of DCs (Wang et al., 2021). Similar to macrophages, DCs have also been found to participate in all stages of atherosclerosis through promoting accumulation of lipids, formation of foam cell, and production of a number of pro-inflammatory cytokines (Branen et al., 2004; Paulson et al., 2010; Koltsova and Ley, 2011). Although lipid uptake, foam cell formation and plaque growth in the artery has been attributed mostly to macrophages, whether the roles of TDP43 regulating atherosclerosis in macrophages and DCs are different and how many foam cells are DC-derived are still not clear and awaits experimental investigation, particularly using lineage-tracking systems.

## Conclusion

We firstly verify the role of TDP43 in macrophage-related inflammatory factors secretion and lipid uptake induced by oxLDL. We also confirmed the promotional effect of TDP43 in atherosclerosis in macrophage-specific knockout mice.

## Data Availability Statement

The raw data supporting the conclusions of this article will be made available by the authors, without undue reservation.

## Ethics Statement

The patients who donated the samples have signed informed consent, and the project was approved by the Ethics Committee of the Ningbo First Hospital. The patients/participants provided their written informed consent to participate in this study. All mouse experiments were conducted after approval by the Ethics Committee of Ningbo First Hospital.

## Author Contributions

NH and XC conceived and designed the experiments. NH, YW, ZX, WZ, CT, ZL, and YH performed the experiments and analyzed the data. All authors wrote the manuscript, read and approved the manuscript.

## Conflict of Interest

The authors declare that the research was conducted in the absence of any commercial or financial relationships that could be construed as a potential conflict of interest.
