# Supplementary figures and images for: TDP43 Exacerbates Atherosclerosis Progression by Promoting Inflammation and Lipid Uptake of Macrophages
Source: Front Cell Dev Biol. 2021 Jul 5;9:687169. doi: 10.3389/fcell.2021.687169 (PMC8287832; doi:10.3389/fcell.2021.687169)

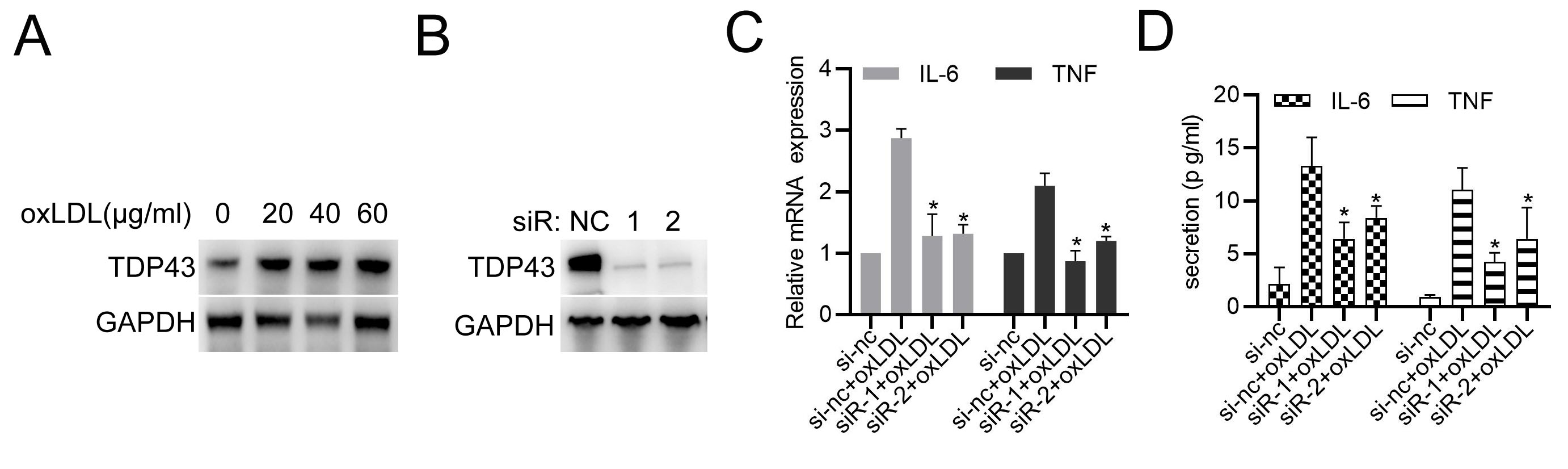

Supplement: Supplementary file 1 [file Image_1.jpeg]

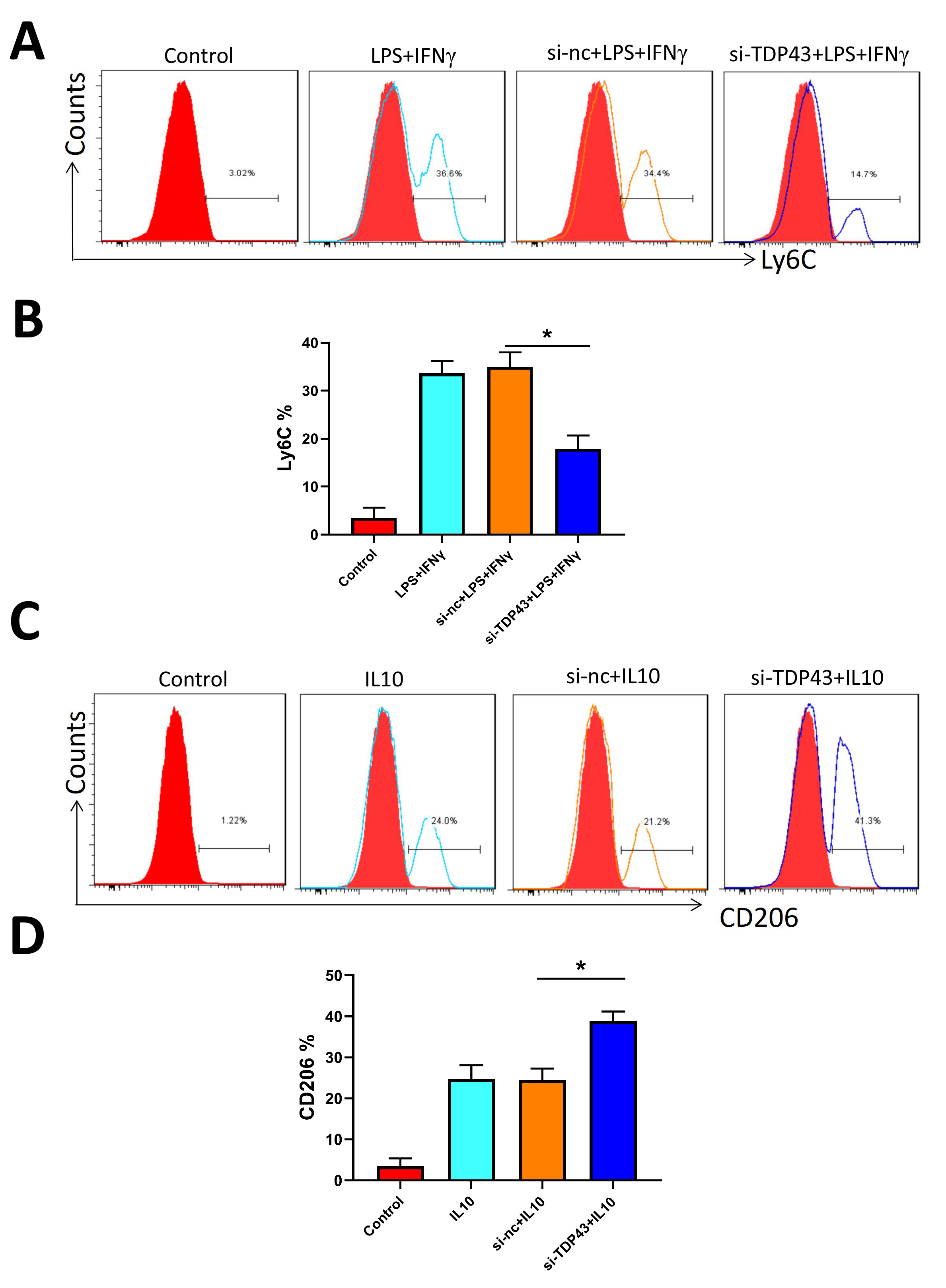

Supplement: Supplementary file 2 [file Image_2.jpeg]
